# Comprehensive Evolutionary and Structural Analysis of the H5N1 Clade 2.4.3.4b Influenza a Virus Based on the Sequences and Data Mining of the Hemagglutinin, Nucleoprotein and Neuraminidase Genes Across Multiple Hosts

**DOI:** 10.3390/pathogens14090864

**Published:** 2025-08-31

**Authors:** Kalpana Singh, Yashpal S. Malik, Maged Gomaa Hemida

**Affiliations:** 1 Department of Bioinformatics, College of Animal Biotechnology, Guru Angad Dev Veterinary and Animal Sciences University, Ludhiāna 141004, India; kalpana.iiita@gmail.com; 2 ICAR-Indian Veterinary Research Institute, Campus Mukteswar, Distt. Nainital 263138, India; malikyps@gmail.com; 3 Department of Veterinary Biomedical Sciences, College of Veterinary Medicine, Long Island University, Brookville, NY 11548, USA

**Keywords:** H5N1, Influenza A virus, clade 2.3.4.4b, hemagglutinin, nucleoprotein, neuraminidase, phylogenetics, selection pressure, molecular dynamics, protein–ligand docking

## Abstract

H5N1 Influenza A virus continues to pose a significant zoonotic threat, with increasing evidence of interspecies transmission and genetic adaptation. Previous studies primarily focused on avian or human isolates, with limited comprehensive analysis of H5N1 evolution across multiple mammalian hosts. Existing molecular surveillance often lags behind viral evolution; this study underscores the necessity for real-time monitoring of ongoing mutations affecting pathogenicity and transmissibility. Our goals are (1) to retrieve and analyze HA, NP and NA gene sequences of H5N1 Influenza A virus from diverse hosts, including humans, poultry and multiple mammalian species, to assess genetic diversity and evolutionary patterns and (2) to evaluate positive selection sites across the three major genes (HA, NP and NA) to determine adaptive mutations linked to host adaptation and viral survival. To achieve these goals, in this study, we considered (78 HA), (62 NP) and (61 NA) gene sequences from diverse hosts, including humans, poultry and multiple mammalian species, retrieved from the NCBI database. Phylogenetic analysis revealed distinct clade formations, indicating regional spread and cross-species transmission events, particularly from avian sources to mammals and humans. Selection pressure analysis identified positive selection across all three genes, suggesting adaptive mutations contributing to host adaptation and viral survival. Homology modeling and molecular dynamics simulations were performed to generate high-quality structural models of HA, NP and NA proteins, which were subsequently validated using multiple stereochemical parameters. Domain analysis confirmed conserved functional motifs, while protein–ligand docking demonstrated stable interactions at conserved binding sites, despite observed residue substitutions in recent isolates. Earlier research concentrated on HA alone; this study integrates HA, NP and NA genes for a broader understanding of viral evolution and adaptation. These findings highlight ongoing evolutionary changes in H5N1 genes that may enhance viral adaptability and pathogenicity, underscoring the need for continuous molecular surveillance and updated antiviral strategies.

## 1. Introduction

Influenza A viruses (IAVs), belonging to the family Orthomyxoviridae, are among the most significant pathogens affecting humans and animals, and cause seasonal epidemics and occasional pandemics that result in substantial morbidity, mortality and economic losses worldwide [1,2,3,4]. Among these, highly pathogenic avian Influenza A(HPAI) H5N1 viruses pose a particularly grave zoonotic threat. Since their first detection in domestic poultry in the late 1990s, H5N1 viruses have spread across Asia, Europe, Africa and the Americas, causing devastating outbreaks in poultry populations, and sporadic but severe infections in humans [5]. The World Health Organization (WHO) reports a case fatality rate exceeding 50% in humans, underscoring the pandemic potential of these viruses should sustained human-to-human transmission occur [6]. The evolution of H5N1 viruses is driven by their segmented RNA genome, which facilitates frequent genetic reassortment and accumulation of point mutations through antigenic drift. These mechanisms enable the virus to evade host immune responses, adapt to new hosts and develop resistance to antivirals [7]. While wild aquatic birds serve as the primary reservoir of IAVs, recent years have witnessed an alarming increase in spillover events to domestic mammals (such as cats, dogs, pigs and cattle) and wildlife, highlighting the virus’s expanding host range [8]. Notably, the emergence and global dissemination of H5N1 clade 2.3.4.4b since 2020 have raised concerns due to its unprecedented spread across continents and its association with widespread infections in avian and mammalian hosts, including recent reports of infections in sea lions, mink and dairy cattle [9]. These events amplify fears of viral adaptation toward efficient mammalian transmission, which could pave the way for the next influenza pandemic [10]. At the molecular level, three viral proteins—hemagglutinin (HA), nucleoprotein (NP) and neuraminidase (NA)—play critical roles in host adaptation and viral fitness. HA mediates receptor binding and membrane fusion, thereby determining host specificity and pathogenicity [11]. The NA protein facilitates viral release from infected cells by cleaving sialic acid residues, influencing transmission dynamics and drug susceptibility. NP forms the ribonucleoprotein complex essential for genome packaging and replication, and mutations in NP have been implicated in host adaptation and immune evasion [12]. Several studies have mainly focused on the HA gene due to its central role in receptor binding and antigenicity; however, there is limited integrated analysis of the combination of the HA alongside the NP and the NA in the context of genetic evolution, structural variation and functional consequences across diverse hosts [13]. Existing literature also exhibits several gaps that impede a comprehensive understanding of H5N1 evolution and its zoonotic potential: Limited cross-host evolutionary insight: most studies have focused on either avian or human isolates, neglecting sequences from other mammalian hosts that increasingly act as intermediate or spillover reservoirs [14]. Inadequate multi-gene approach: research on HA is abundant, but NP and NA, which influence replication efficiency and drug resistance, are often overlooked in combined analyses [15]. Lack of structural-functional correlation: while sequence-based analyses of H5N1 genes are available, few studies incorporate structural modelling, molecular dynamics (MD) simulations and ligand-binding assessments to evaluate the functional implications of adaptive mutations [16]. Insufficient data on contemporary isolates: rapid viral evolution necessitates real-time surveillance of recent isolates from multiple species, yet integrated molecular and structural studies using updated datasets remain scarce. Addressing these gaps is crucial for predicting the evolutionary trajectory of H5N1, guiding the development of antiviral drugs and informing vaccine design strategies. The present study aims to provide a comprehensive evolutionary and structural characterization of H5N1 clade 2.3.4.4b isolates using three key viral genes—HA, NP and NA—retrieved from multiple species, including humans, poultry and various domestic mammals [17]. Specifically, we conducted phylogenetic analyses to elucidate genetic relationships and identify patterns of interspecies transmission, assess selection pressures acting on HA, NP and NA genes to detect signatures of adaptive evolution, predict and validate three-dimensional structures of these proteins using homology modelling and molecular dynamics simulations to understand the structural consequences of mutations [18], and analyze conserved domains and evaluate protein–ligand interactions through molecular docking to determine the impact of residue substitutions on receptor binding and antiviral susceptibility. By integrating evolutionary, structural and functional analyses, this study provides valuable insights into the mechanisms underlying H5N1 adaptability, thereby enhancing preparedness for future outbreaks and informing the development of effective countermeasures.

## 2. Materials and Methods

### 2.1. Retrieval of Gene Sequences and Analysis

A total of 78, 62 and 61 gene sequences of HA, NP and NA proteins, respectively, from various isolates of H5N1 strain of Influenza A virus clade 2.4.4.4b from varied hosts such as human, chicken, dog, horse, cattle, cat, pig and goat (NP gene sequences from horse were not available) were retrieved from the NCBI nr-nucleotide database. These retrieved gene sequences were renamed with their accession ID, host name, country of isolation and isolate ID. Further, sequence similarity and coverage were assessed for all sequences. The details of the used sequences for each protein are listed in Supplementary Tables S1–S4. 

### 2.2. Phylogenetic and Selection Pressure Analyses of HA, NP and NA Genes

Multiple sequence alignments were performed for sequences of HA, NP and NA genes using Clustal Omega [19]. Then, phylogenetic analysis was performed using PHYLIP v3.698 [20] with the neighbor-joining algorithm. Then, phylogenetic trees were visualized for gene sequences of HA, NP and NA proteins using MEGA11 vBeta [21]. Further, selection pressure analysis was done for both HA, NP and NA gene sequences at gene and codon levels using HYPHY/Datamonkey v2.5 [22] at the threshold of *p* ≤ 0.1. 

### 2.3. Protein Sequence Retrieval and Phylogenetic Analysis of HA, NP and NA Proteins

Protein coding sequences of all sequences of HA, NP and NA genes were retrieved from NCBI (https://www.ncbi.nlm.nih.gov/core/ncbi_index/, accessed on 31 July 2025) for all the isolates from hosts such as horse (NP protein sequences from horse were not available), dog, cat, human, chicken, goat, pig and cattle. Further, multiple sequence alignment was performed for protein sequences of HA, NP and NA genes using Clustal Omega v1.2.4. Then, phylogenetic analysis was performed using PHYLIP v3.698 with the neighbor-joining algorithm. Then, phylogenetic trees were visualized for protein sequences of HA, NP and NA genes using MEGA11 vBeta [21].

### 2.4. Structure Prediction and Assessment of HA, NP and NA Proteins

The structures of the (HA, NP and NA) proteins were predicted using the protein MODELLER v10.7 (Protein Modeling with Modeller: A Comprehensive Guide-Omics tutorials) [23], after template structure selection for HA, NP and NA proteins using BLASTp (https://blast.ncbi.nlm.nih.gov/Blast.cgi?PROGRAM=blastp&PAGE_TYPE=BlastSearch&LINK_LOC=blasthome, accessed on 31 July 2025) [24]. Further, the quality of the structure was assessed using ERRAT (https://www.doe-mbi.ucla.edu/errat/, accessed on 31 July 2025) [24], ProSA (https://bio.tools/prosa-web, accessed on 31 July 2025) [25] and PROCHECK (https://www.ebi.ac.uk/thornton-srv/software/PROCHECK/, accessed on 31 July 2025) [26] for each protein. Domains were also assessed from the predicted structures using InterPro (https://www.ebi.ac.uk/interpro/, accesed on 31 July 2025) [27].

### 2.5. MD Simulation of Predicted Structures of HA, NP and NA Proteins

The GROMACS v2025.2 was used for MD simulation to get the more stable optimized structures of HA, NP and NA proteins within the cellular environment. First, the OPLS force field was selected for all the atoms of the proteins, along with the SPC/E water model with a 1000 kJ mol^−1^ nm^−2^ force constant (kpr) to mimic the cellular aqueous medium. To simulate the aqueous medium, each protein was placed in the center of a cubic box filled with the water molecules at least 1.0 nm away from the box edge, along with the solvent configuration spc216.gro. The volume of the cubic box was optimized to ensure the complete coverage of the solvent over each protein. Further, 7 Na^+^, 12 Cl^−^ and 6 Na^+^ ions were added by replacing a similar number of solvent molecules to neutralize the −7, 12 and −6 charges of the (HA, NP and NA) proteins, respectively. Before performing molecular dynamic simulations, energy minimization of the protein–solvent system was performed to avoid steric clashes or inappropriate geometry using steepest descent minimization up to a maximum force < 1000.0 kJ mol^−1^ nm^−1^ for 50,000 maximum minimization steps. Further, to begin fundamental dynamics, equilibration of the solvent and ions around each protein was done in two phases. The first phase was conducted under a constant NVT (number of particles, volume and temperature) ensemble to stabilize the temperature of the protein–solvent system using a modified Berendsen thermostat for 100 ps. The second phase was conducted under a constant NPT (number of particles, pressure and temperature) ensemble to stabilize the pressure of the system using the Parrinello–Rahman barostat and modified Berendsen thermostat for 100 ps. After achieving the desired temperature and pressure of the well-equilibrated protein–solvent system, MD simulation was performed for each protein for 1000 ps. The final optimized structure of each protein was utilized for further protein–ligand docking.

### 2.6. Docking of the H5N1 Clade 2.3.4.4b (HA, NP and NA) Proteins with Their Ligand Receptors

Information on natural ligands of HA, NP and NA proteins was taken from the published literature, such as the sialated glycan receptor (NAG-GAL-SIA) of the HA protein [13,28,29], RNA sequence of the NP protein [30] and sialic acid (SIA) of the NA protein [31]. Structures of ligands were drawn using SwissADME online tools (SwissADME) and further, hydrogen atoms were added and charges were assigned to optimized structures of HA, NP and NA proteins and hydrogen atoms were added to their respective ligands using PyMol v3.1., accessed on 31 July 2025. Further, molecular docking was performed for each protein with its respective ligand to get the information of the binding site in terms of the least binding score and binding-site residues using the CD-Dock2 server (https://cadd.labshare.cn/cb-dock2/, accessed on 31 July 2025) [32] utilizing a template-based bling docking approach. CD-Dock2 follows a procedure that combines curvature-based cavity detection with Auto Dock Vina-based molecular docking.

## 3. Results and Discussion

### 3.1. Sequence Analysis of HA, NP and NA Genes

Details of a sum of 78, 62 and 61 sequences retrieved from NCBI for HA, NP and NA genes, respectively, are provided in Supplementary Tables S5, S6 and S7, respectively. With respect to the PP755366.1/goat/Minnesota/24-007234-006/2024 HA gene, another 77 HA gene sequences have sequence similarity up to 85% and coverage up to 99.8%. Out of 78 gene sequences of the HA protein, 10, 18, 11, 6, 4, 9, 3 and 17 sequences were extracted from cattle, human, cat, goat, horse, swine, dog and chicken hosts, respectively, from USA, Japan, South Korea, Thailand, Indonesia, Viet Nam, China, Nigeria, Germany, Hong Kong and Bangladesh between 2001 to 2025. Similarly, out of 62 gene sequences of NP protein, sequences were extracted from cattle (4), humans (11), cats (6), goats (2), horses (2), pigs (4), dogs (1) and chickens (32), respectively, from Germany, Sudan, Burkina Faso, China, USA, Indonesia, Thailand, Cambodia, Kuwait, Poland, Russia, Ivory Coast, Israel, Scotland, Cameroon, Bangladesh, Bhutan, Nepal, Pakistan, Afghanistan, South Korea, Malaysia, Nigeria, Laos, Ghana, Philippines, India, Egypt, England, Viet Nam, France, Chile, Canada and Hong Kong. There was sequence similarity of up to 82% and coverage of up to 95.5% among NP gene sequences with respect to the NP gene sequence of PV124897.1/cattle/Hong Kong/RG-DelNS1-p10/2024. Similarly, there was sequence similarity of up to 77.4% and coverage of up to 94.8% among NA gene sequences with respect to the NA gene sequence of PV649629.1/chicken/NY/25-010935-008-original/2025. Out of 61 gene sequences of NA protein, 8, 14, 6, 3, 3, 12, 2 and 13 sequences were extracted from cattle, human, cat, goat, horse, swine, dog and chicken hosts, respectively.

### 3.2. Phylogenetic Analysis of HA, NP and NA Genes

**Figure 1:**
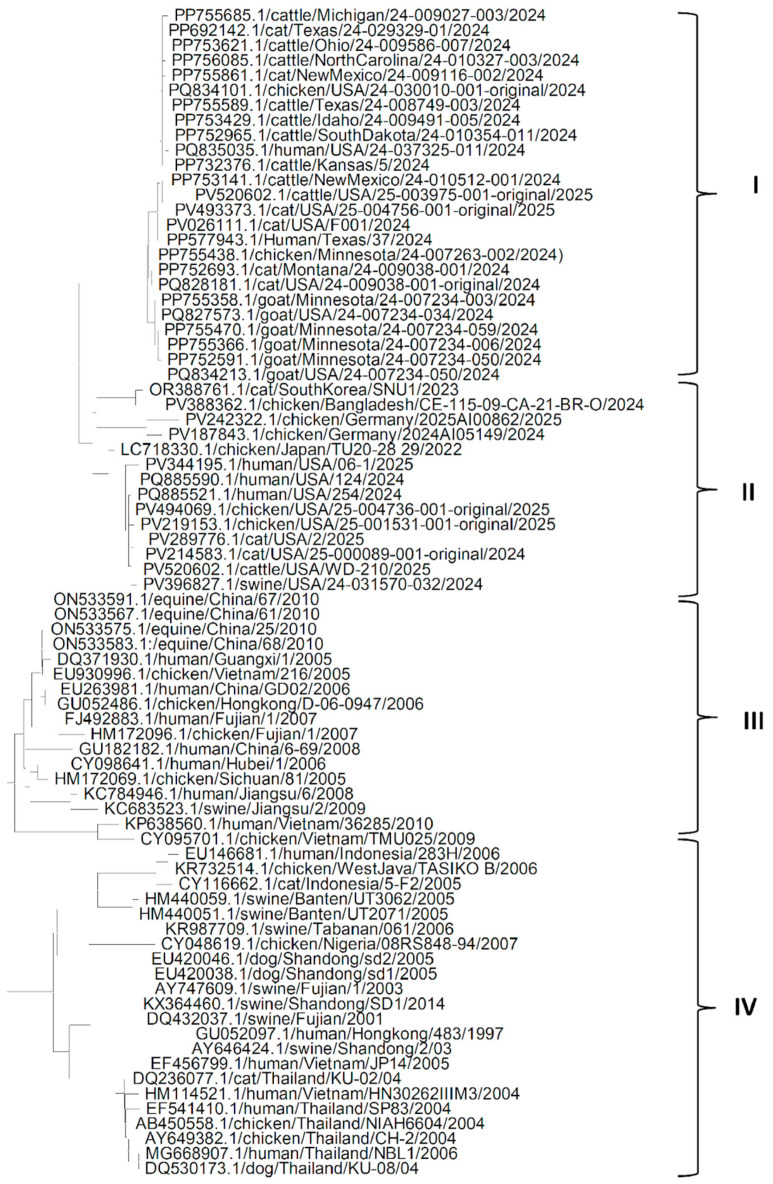
Phylogenetic tree for the HA gene of H5N1 Influenza A virus clade 2.3.4.4b from various hosts.

The phylogenetic analysis of the HA gene (Figure 1) shows that the genes from different isolates of the H5N1 Influenza virus could be grouped into four clades. It can be seen from clade I that the HA gene from American isolates of the H5N1 Influenza virus from different hosts such as goat, cattle, human, cat and chicken, are more closely related to each other, which may suggest that H5N1 Influenza virus might have spread through humans to other domestic host species living with the human population. A similar pattern is seen in clade II, suggesting that the H5N1 isolates might have reached the USA via an affected human from H5N1-infected chickens in Europe, and H5N1 isolates might have reached Europe through affected chickens from Asia. Clades III and IV suggest that in Asia, the H5N1 influenza virus might have reached human and other domestic animal species populations through chickens. It can also be seen that HA genes from most of the isolates from China are similar to each other and might have spread from chickens to other mammalian species. It is also evident from the phylogenetic tree of the HA gene that the HA gene from most of the isolates of different hosts from Asian countries other than China might have spread from China and are more like each other. Another important observation from Figure 1 is that the HA gene sequences of isolates in clades I and II were recently isolated and sequenced and were like each other, regardless of the host of the isolates. However, the HA gene sequences of isolates in clades III and IV were older. This suggests that older isolates were somewhat distinct from recent isolates.

**Figure 2:**
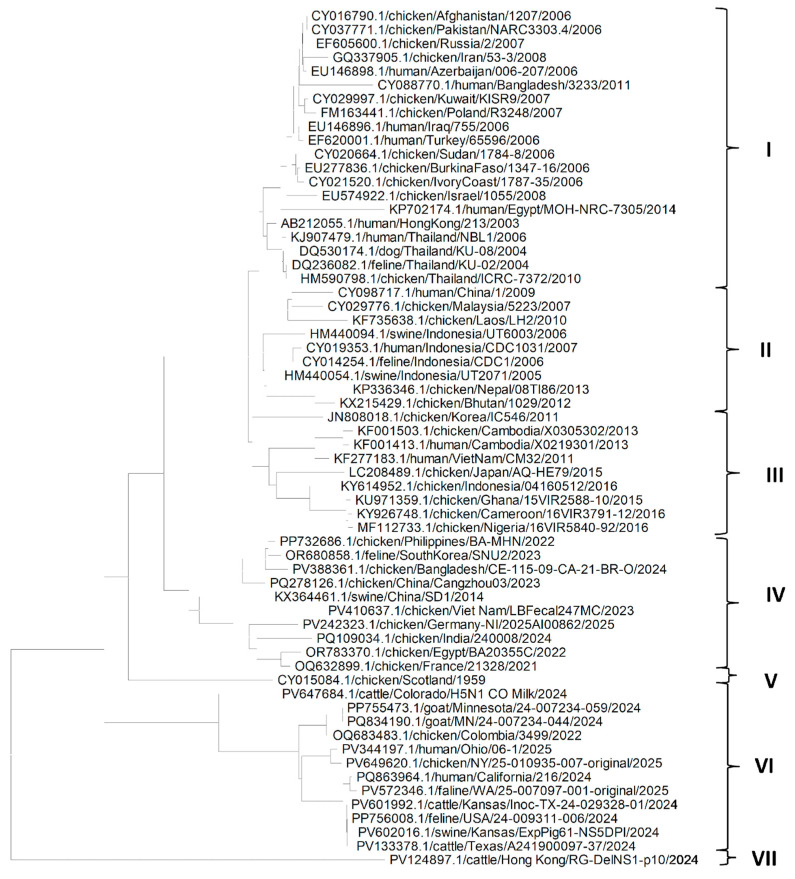
Phylogenetic tree for the NP gene of the H5N1 Influenza A virus clade 2.3.4.4b from various hosts.

The phylogenetic analysis of the NP gene (Figure 2) shows that NP genes from different isolates of the H5N1 Influenza virus could be grouped mainly in seven clades. Clade I is the largest, suggesting that H5N1 isolates may have migrated from Asia to the Middle East, and via Russia to Europe. It also indicates that the migration of H5N1 infection could have occurred from chickens to different domestic host species in Asia and other countries. Similarly, a conclusion could be derived from clades II and III that the migration of H5N1 infection could have occurred from chickens to different domestic host species in Asia. Clade III also suggests the migration of H5N1 from Asia to Africa via infected chickens. Clade IV suggests migration of H5N1 from Asia to Europe via Egypt. It can be seen from the clade VI that the NP gene from American isolates of the H5N1 Influenza virus from different hosts such as goat, cattle, human, cat, swine and chicken are more closely related to each other, which may suggest that H5N1 Influenza virus might have spread through humans to other domestic host species living with the human population. Clades V and VII consist of only one isolate of H5N1 each—from chickens and cattle from Scotland and Hong Kong, respectively. It can also be seen in Figure 1, that except for KX364460.1/swine/Shandong/SD1/2014, NP gene sequences of isolates in clades IV, VI and VII were isolated and sequenced recently, and were like each other irrespective of the host of the isolates; however, the NP gene sequences of isolates of other clades were older. This also suggests that older isolates were somewhat distinct from recent isolates.

**Figure 3:**
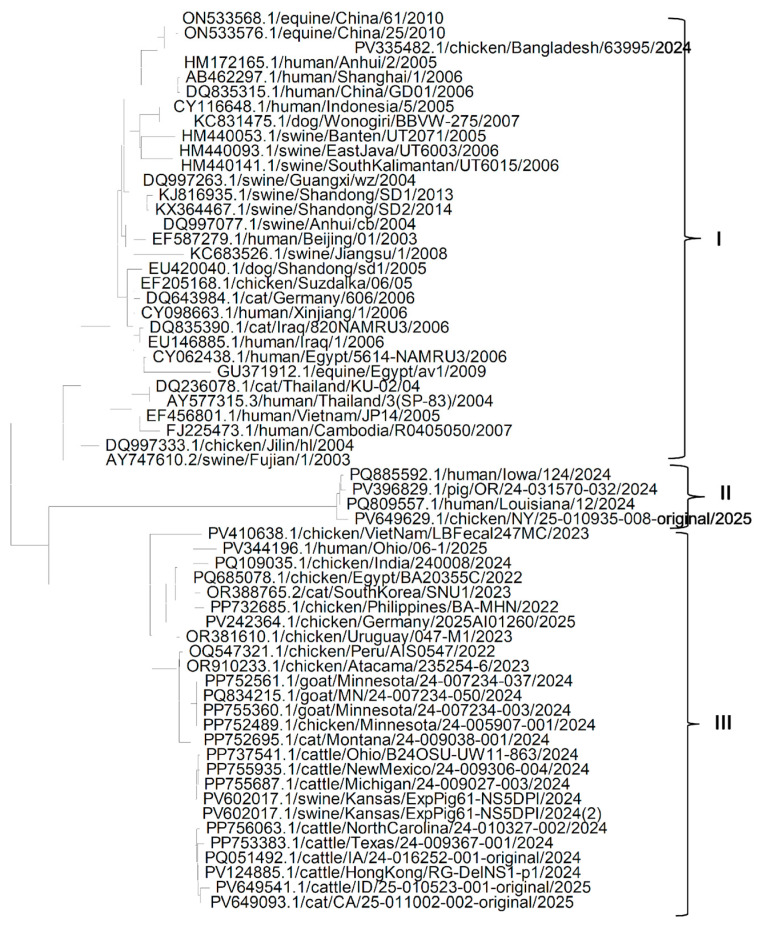
Phylogenetic tree for the NA gene of the H5N1 Influenza A virus clade 2.3.4.4b from various hosts.

Phylogenetic analysis of the NA gene (Figure 3) found that the NA gene sequences of various isolates of H5N1 Influenza A virus could be grouped into three clades. Clade I is the largest and Clade II is the smallest. Clade I suggests that most of the Asian isolates are similar to each other and are older, irrespective of the host, in comparison to isolates in clades II and III. It also suggests that in Asia, the H5N1 influenza virus might have spread from chickens to humans and from humans to other domestic species, though in Middle East countries like Iraq and Egypt, it might have been transmitted by an infected human and spread to other domestic species reared alongside the human population. It can be seen from clade II that the NA gene from various isolates from chicken spread to human and pig populations. Clade III suggests that different isolates of H5N1 Influenza A virus might have been introduced to North and South America from infected chickens from Viet Nam. It is also very interesting to see from clade III that there must have been a fresh spread of new strains/isolates of the H5N1 Influenza virus from Asia to Europe, the Middle East, North America and South America, which could be concluded based on the similarity of the NA genes from recent isolates, irrespective of host and country of isolation.

### 3.3. Selection Pressure Analysis of HA, NP and NA Genes

Selection pressure analysis (Table 1) suggested that there was positive selection pressure on all three HA, NP and NA genes, though a sum of 3, 2 and 13 codons of HA, NP and NA genes, respectively (see Table 2 for details), were under negative selection pressure. Overall positive selection on the HA, NP and NA genes suggests that new modifications occurred over the time, favored by the nature for the better survival of the H5N1 Influenza virus, which helped it to spread from avian to human hosts and other mammal species which were not the natural hosts of the Influenza virus, but over the time it succeeded in increasing its pathogenicity and invaded all domestic species reared with human populations. 

### 3.4. Phylogenetic Analysis of the HA, NP and NA Proteins of the H5N1 Clade 2.3.4.4b

**Figure 4:**
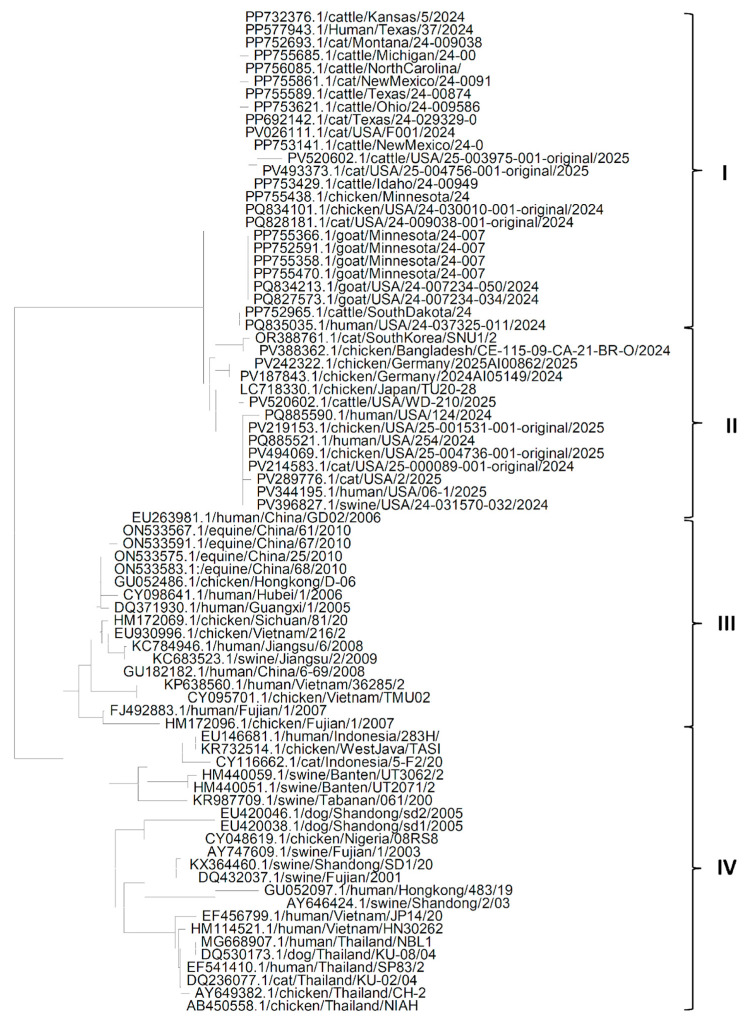
Phylogenetic tree for the HA protein of the H5N1 Influenza A virus clade 2.3.4.4b from various hosts.

The phylogenetic tree (Figure 4) of HA protein sequences of various isolates of the H5N1 Influenza virus shows that there are mainly four clades. Clade I is the largest clade and has HA protein sequences of isolates from the USA from cats, chickens, humans, goats and cattle [33,34,35]. It shows high sequence similarity among HA protein sequences of isolates from the USA, irrespective of the host. Clade II has sequences of isolates from Asia (South Korea, Japan and Bangladesh), Europe (Germany) and the USA from swine, cats, cattle, chickens and humans, which suggests that the H5N1 Influenza virus might have reached the USA from Asia via Europe. Clade III contains HA sequences of isolates from East Asia (China, Hong Kong and Viet Nam) extracted from human, equine, chicken and swine hosts. Clade IV also contains HA sequences of isolates from East Asia (China, Hong Kong, Indonesia, Thailand and Viet Nam) extracted from humans, chickens, dogs, cats, swine, humans, chickens, except one chicken sequence from Nigeria, which suggests that the H5N1 influenza virus might have reached Africa via Asia. All HA sequences of all isolates of the H5N1 Influenza virus in clades I and II are from isolates recently extracted, in comparison to isolates in clade III and IV, which suggests that the new isolates prevailing in the USA might have reached the USA from new variants in Asia that might be more adaptable and able to invade hosts in new environmental conditions.

**Figure 5:**
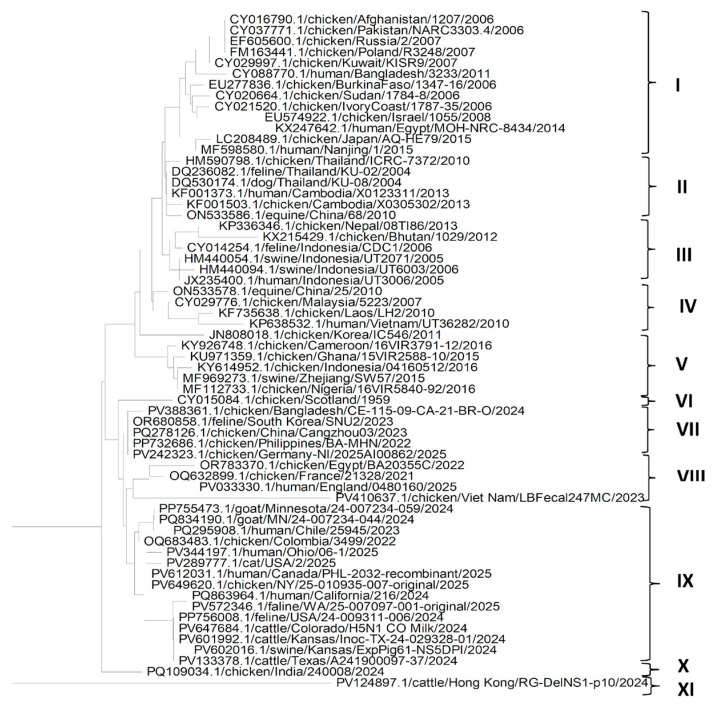
Phylogenetic tree for the NP protein of the H5N1 Influenza A virus clade 2.3.4.4b from various hosts.

The phylogenetic analysis of the NP protein (Figure 5) shows that NP genes from different isolates of the H5N1 Influenza virus could be grouped mainly into eleven clades. Clades VI, X and XI includes only one sequence each from Scotland and India extracted from chickens and from Hong Kong extracted from cattle. Clade I has sequences of H5N1 isolates from East Asia (Pakistan, Bangladesh, Afghanistan, Russia, Japan and China), the Middle East (Kuwait, Israel and Egypt), Africa (Sudan, Burkina Faso and Ivory Coast) and Europe (Poland) extracted mainly from chickens, and a few from humans. This suggests that H5N1 isolates may have infected humans from chickens and migrated from East Asia to the Middle East and Africa. Clade II includes sequences from East Asia (China, Cambodia, Japan and Thailand) from chicken, cat, dog, human and equine hosts. Clade III includes sequences of isolates from Asia (Nepal, Bhutan and Indonesia) extracted from chickens, cats and swine. Similarly, clade IV has sequences of isolates from Asia (China, Malaysia, Laos and Viet Nam) extracted from equine, human and chicken species. It could be determined from clades II, III and IV that the migration of H5N1 infection could have occurred from chickens to different domestic host species in Asia. Clade V includes sequences of isolates from Asia (China, South Korea and Indonesia) and Africa (Ghana, Nigeria and Cameroon) extracted from chickens and swine. This might suggest the migration of the H5N1 influenza virus from Asia to Africa via infected chickens. Clade VII includes sequences of isolates from Asia (China, Bangladesh, South Korea and the Philippines) and Europe (Germany) extracted from cats and chickens. Clade VIII includes sequences from Asia (Viet Nam), the Middle East (Egypt) and Europe (England and France). This suggests the migration of the H5N1 Influenza virus from Asia to Europe and the Middle East. Clade IX includes sequences of NP protein isolates from North America (USA and Canada) and South America (Chile and Colombia) from different hosts such as goat, cattle, human, cat, swine and chicken. It can be seen that the sequences of clades VII, VIII and IX are more recent and closely related to each other than the older sequences of clades I, II, III, IV and V.

**Figure 6:**
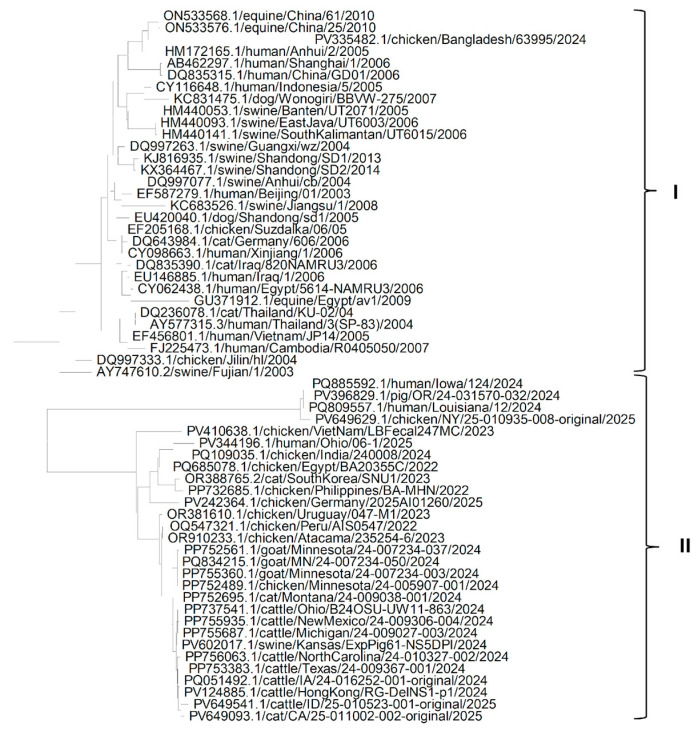
Phylogenetic tree for the NA protein of H5N1 Influenza A virus from various hosts.

It could be seen from the phylogenetic tree (Figure 6) of sequences of the NA protein of isolates of the H5N1 Influenza virus that all sequences could be grouped mainly into two clades. Clade I includes all the older sequences of isolates from Asia (China, Indonesia, Thailand, Indonesia, Cambodia and Viet Nam), the Middle East (Egypt and Iraq) and Europe (Germany) extracted from chicken, human, equine, cat, dog and swine hosts, except for one sequence of an isolate from Bangladesh extracted in 2024. Clade II includes all sequences of newer isolates extracted recently from North America, South America (Peru, Uruguay and Atacama), Asia (India, Viet Nam, South Korea, Philippines and Hong Kong) and the Middle East (Egypt) extracted from cats, chickens, pigs, goats, humans and cattle. This suggests that newer isolates of the H5N1 Influenza virus might have developed adaptations for newer climatic conditions (because they are different from the older isolates, as both fall in different clades) and spread to North America and South America from Asia. 

### 3.5. Protein Structure Prediction, MD Simulation and Domain Analysis of the HA, NP and NA Proteins

**Figure 7:**
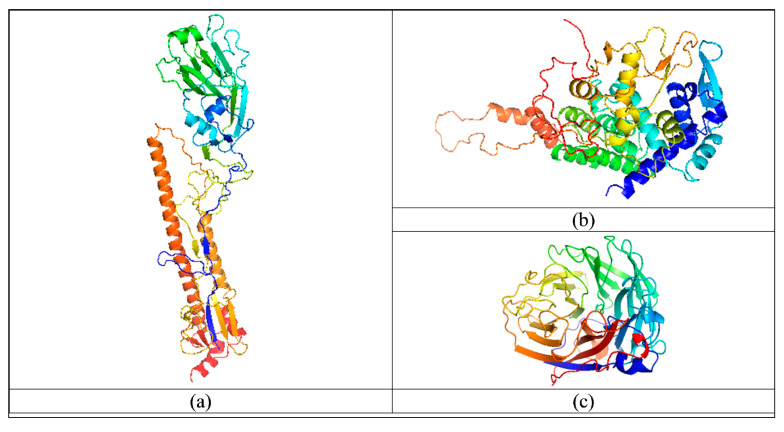
Predicted structures of HA (**a**), NP (**b**) and NA proteins (**c**).

First, 6pcx.1.A protein structure was selected as a template structure with 91.87% sequence identity and 89% coverage with the target sequence of the HA protein for the structure prediction using homology modelling. Similarly, 2q06.A, which has a sequence identity of 97.39% and coverage of 100% with the target sequence of the NP protein, was selected as a template for the structure prediction. 5hug.1.A protein sequence, which has 96.12% sequence identity and 88% coverage with the target sequence of the NA protein, was selected as a template structure for structure prediction. The best model obtained from homology modelling was selected for each protein. Predicted structures of HA, NP and NA proteins are shown in Figure 7a, Figure 7b and Figure 7c, respectively. Further, quality assessment was performed for all proteins by PROCHECK (Ramachandran score), ERRAT, VERIFY3D, QMEAN and Z-score and assessed as good predicted structures with higher % of allowed region, higher ERRAT score, higher VERIFY3D score, QMEAN score close to 0 and Z-score within range, respectively (details of pre-MD-simulation scores are given in Table 3). Further, predicted structures were optimized using MD simulation and assessed as mentioned above for pre-MD-simulation structure assessment using Ramachandran score, ERRAT, VERIFY3D, QMEAN, Z-score, etc.; results are shown in Table 4 as post-MD-simulation scores, and show the significant improvement in structure post-MD-simulation reflected in the improvement in different scores.

Additionally, domains in all three proteins were analyzed using InterPro. The Hemagglutinin domain belonging to viral capsid protein domain superfamily and HA1 A/B domain belonging to Hemagglutn_HA1_a/b domain superfamily were found in HA protein, as reported in HA protein of Influenza A virus isolates. However, no specific domain was found in the NP protein and it contained only a small, low-complexity region in positions 177 to 187. The Sialidase Influenza A/B (IPR033654) domain, belonging to the Sialidase domain superfamily, was identified in the NA protein, consistent with other Influenza A virus isolates.

### 3.6. Molecular Docking of HA, NP and NA Proteins with Their Ligand

**Figure 8:**
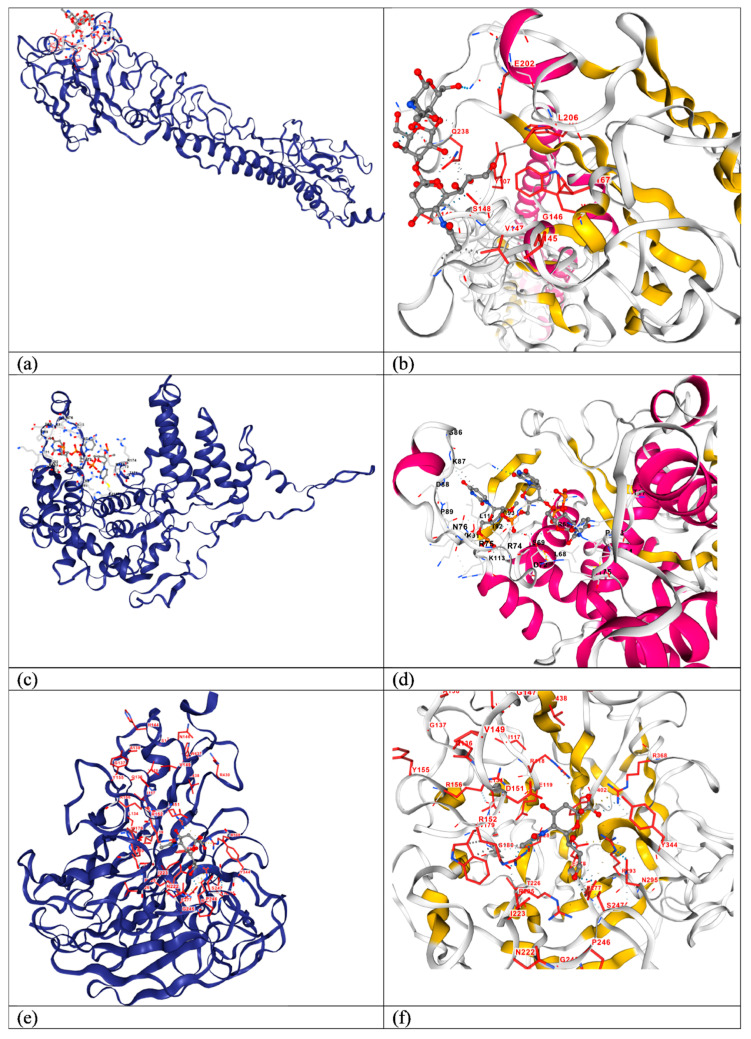
Ligand (shown in stick–ball model)-binding site of the HA protein (shown in cartoon model) in full (**a**) and closer (**b**) views; ligand (stick–ball model)-binding site of NP protein (cartoon model) in full (**c**) and closer (**d**) views; ligand (stick–ball model)-binding site of NA protein (cartoon model) in full (**e**) and closer (**f**) views.

Template-based blind molecular docking was performed for all three proteins with their respective ligands, the results of which are provided in Table 4, including information on ligand, template used, docking score and ligand-binding-site residues. Figure 8 shows the ligand-binding site in the protein (shown in a cartoon model) in full view and a closer view, along with the ligand (shown in a stick–ball model). Ligand-binding-site residues derived using molecular docking were found to be conserved throughout 78 sequences of HA protein from different isolates, irrespective of varied hosts such as human, chicken, dog, horse, cattle, cat, pig and goat, except S149, A150 and D199, which were found substituted with A149, S150/V150 and N199. In HA protein sequences of recent isolates, as grouped in clades I and II (Figure 4), the binding-site residues A149, N199 (S199 in PV388362.1/chicken/Bangladesh/CE-115-09-CA-21-BR-O/2024), Q234 substituted S149, D199 and K234, respectively, were found in older isolates grouped in clade III and IV. These substitutions might have helped the H5N1 Influenza virus adapt to new environmental conditions and facilitate its spread to new geographic locations like North and South America. Binding-site residue A150 was found substituted with S150 and V150 in the HA protein sequence of HM440059.1/swine/Banten/UT3062/2005 and CY098641.1/human/Hubei/1/2006, respectively. The binding-site residue Q238 was found substituted with L238 in the HA protein sequence of CY116662.1/cat/Indonesia/5-F2/2005. Ligand-binding-site residues derived using molecular docking were found to be conserved throughout 62 sequences of the NP protein from different isolates, irrespective of varied hosts such as human, chicken, dog, cattle, cat, pig and goat, except S68, H82 and L136, which were substituted with Y68, R82 and I136, respectively. In the NP sequence of PV410637.1/chicken/Viet Nam/LBFecal247MC/2023, binding-site residue H82 was found substituted by R82 and in the NP protein sequence of KX215429.1/chicken/Bhutan/1029/2012, binding-site residue S68 was found substituted by Y68. In the NP protein sequence of KX215429.1/chicken/Bhutan/1029/2012, binding-site residue L136 was found substituted by I136. Ligand-binding-site residues derived using molecular docking were found to be conserved throughout 61 sequences of NA protein from different isolates, irrespective of varied hosts such as human, chicken, dog, horse, cattle, cat, pig and goat.

## Figures and Tables

**Table 1:** List of the non-synonymous/synonymous rate ratio for the HA, NP and NA genes.

Gene	No. of Sequences	No. of Codons	No. of Codons Under Selection	Non-Synonymous/Synonymous Rate Ratio	Selection Pressure
HA gene	78	530	3	2.7856	Positive
NP gene	61	439	2	2.4435	Positive
NA gene	62	435	13	2.4312	Positive

**Table 2:** Selection pressure on codons of HA, NP and NA genes.

Protein	Codon No.	S	N	dS	dN	Selection Pressure
HA	151	0.000	6.000	0.000	3.000	Positive
	191	3.000	1.000	3.752	0.454	Negative
	487	2.000	0.000	2.499	0.000	Negative
NP	10	0	4	0	2.507	Positive
	138	1	9	0	9	Negative
NA	17	1	5	0.678	3.538	Positive
	45	6	5	7.237	2.313	Negative
	91	0	6	0	2.979	Positive
	105	0	6	0	3.02	Positive
	112	0	7	0	3.464	Positive
	175	3	0	3	0	Negative
	181	0	6	0	2.986	Positive
	235	0	8	0	4.102	Positive
	271	3	1	4.117	0.44	Negative
	347	1.5	5.5	0.917	4.03	Positive
	348	0	9	0	4.287	Positive
	352	0	6	0	2.997	Positive
	402	0	6	0	3	Positive

N = non-synonymous sites; S = synonymous sites; dN = rate of non-synonymous sites; dS = rate of synonymous sites.

**Table 3:** Various quality assessment scores of predicted structures of HA, NP and NA proteins: pre- and post-MD-simulation.

Proteins	Assessment Scores	Pre-MD-Simulation	Post-MD-Simulation
HA	Procheck	92.06 (Ramachandran score)	94.79 (Ramachandran score)
		79.1% most favored core	88.7% most favored core
		18.7% additional allowed region	9.7% additional allowed region
		1.6% generously allowed region	0.9% generously allowed region
		0.7% disallowed region	0.7% disallowed region
	ERRAT	91.9913	92.5926
	Verify3D	78.64%	79.44%
	QMEAN	0.71	0.78
	ProSA	−9.8	−9.5
NP	Procheck	89.02 (Ramachandran score)	91.75 (Ramachandran score)
		85.4% most favored core	86.1% most favored core
		12.9% additional allowed region	12.4% additional allowed region
		1.4% generously allowed region	1.2% generously allowed region
		0.2% disallowed region	0.2% disallowed region
	ERRAT	90.9548	96.1276
	Verify3D	84.21%	82.32%
	QMEAN	0.69	0.79
	ProSA	−9.7	−9.36
NA	Procheck	91.53 (Ramachandran score)	94.82 (Ramachandran score)
		76.9% most favored core	86.7% most favored core
		20.4% additional allowed region	13.0% additional allowed region
		2.8% generously allowed region	0.3% generously allowed region
		0.0% disallowed region	0.0% disallowed region
	ERRAT	82.9913	89.0957
	Verify3D	78.64%	84.75%
	QMEAN	0.88	0.93
	ProSA	−5.4	−5.15

**Table 4:** Details and results of molecular docking of HA, NP and NA proteins with their respective ligands.

Protein	Ligand	Template	Docking Score	Binding-Site Residues
HA	NAG-GAL-SIA (N-acetyl-alpha-neuraminic acid-(2-3)-beta-D-galactopyranose-(1-4)-2-acetamido-2-deoxy-beta-D-glucopyranose)	1hge	−2.8	V147 S148 A149 A150 P157 W165 N198 N199 E202 L206 Q234 G237 Q238
NP	RNA	7dxp	−7.5	Q58 I61 T62 R65 M66 L68 S69 A70 D72 E73 R74 R75 N76 H82 G86 K87 D88 P89 K90 K91 T92 G93 G94 P95 L108 L110 K113 R117 L136 M137 H140 T171 L172 P173 R174 R175 S367
NA	SIA (N-ACETYL-ALPHA-NEURAMINIC ACID)	4gzq	−5.7	R118 E119 D151 R152 R156 W179 S180 R225 E228 S247 E277 E278 R293 N295 Y344 G345 R368 Y402

## Data Availability

All data will be available upon request to the corresponding author.

## Supplementary Materials

The following supporting information can be downloaded at: https://www.mdpi.com/article/10.3390/pathogens14090864/s1. Table S1: HA of 279 isolates of H5N1 clade 2.3.4.4b; Table S2: NA of 79 isolates of H5N1 2.3.4.4b Chicken; Table S3: NP of 79 isolates of H5N1 2.3.4.4b; Table S4: M2 of 79 isolates of H5N1 2.3.4.4b; Table S5: HA gene sequences of 78 isolates of H5N1 clade 2.3.4.4b; Table S6: NP gene sequences of 62 isolates of H5N1 clade 2.3.4.4b; Table S7: NA gene sequences of 61 isolates of H5N1 clade 2.3.4.4b.

## Abbreviations

The following abbreviations are used in this manuscript:

HA	Hemagglutinin
NA	Neuraminidase
NP	Nucleoprotein
IAV	Influenza A virus
HPAI	Highly pathogenic avian influenza
WHO	World Health Organization
MD	Molecular dynamics
NCBI	National Center for Biotechnology Information
NVT	Constant number of particles, volume and temperature
NPT	Constant number of particles, pressure and temperature
OPLS	Optimized potentials for liquid simulations
SPC/E	Extended simple point charge (water model)
BLASTp	Basic Local Alignment Search Tool for Proteins
ProSA	Protein Structure Analysis
QMEAN	Qualitative Model Energy Analysis
CD-Dock2	Curvature Detection-based Docking Tool
NAG-GAL-SIA	N-acetyl-alpha-neuraminic acid-(2-3)-beta-D-galactopyranose-(1-4)-2-acetamido-2-deoxy-beta-D-glucopyranose
SIA	N-acetylneuraminic acid (Sialic acid)
