# Supplementary material for: Comprehensive Evolutionary and Structural Analysis of the H5N1 Clade 2.4.3.4b Influenza a Virus Based on the Sequences and Data Mining of the Hemagglutinin, Nucleoprotein and Neuraminidase Genes Across Multiple Hosts
Source: Pathogens. 2025 Aug 31;14(9):864. doi: 10.3390/pathogens14090864 (PMC12472945; doi:10.3390/pathogens14090864)
Supplement: Supplementary file 1 [file pathogens-14-00864-s001.zip › Supp Table S5,S6,S7.pdf]

Table S5: HA gene sequences of 78 isolates of H5N1 clade 2.3.4.4b

S.No.	Sequence detail (GenBank ID/Host/Place/Isolate ID/Year)	Coverage (%)	Identity (%)
1	PP755366.1/goat/Minnesota/24-007234-006/2024	100.0	100
2	PP752591.1/goat/Minnesota/24-007234-050/2024	100.0	100
3	PQ834213.1/goat/USA/24-007234-050/2024	100.0	100
4	PP755358.1/goat/Minnesota/24-007234-003/2024	100.0	99.9
5	PQ827573.1/goat/USA/24-007234-034/2024	100.0	99.9
6	PP755470.1/goat/Minnesota/24-007234-059/2024	100.0	99.9
7	PP752693.1/cat/Montana/24-009038-001/2024	100.0	99.6
8	PQ828181.1/cat/USA/24-009038-001-original/2024	100.0	99.6
9	PP755438.1/chicken/Minnesota/24-007263-002/2024)	100.0	99.6
10	PV520602.1/cattle/USA/25-003975-001-original/2025	100.0	98.8
11	PP732376.1/cattle/Kansas/5/2024	100.0	97.1
12	PV493373.1/cat/USA/25-004756-001-original/2025	100.0	99.2
13	PP577943.1/Human/Texas/37/2024	100.0	96.7
14	PP753141.1/cattle/New Mexico/24-010512-001/2024	100.0	99.4
15	PV026111.1/cat/USA/F001/2024	100.0	95.4
16	PQ835035.1/human/USA/24-037325-011/2024	100.0	99.3
17	PP752965.1/cattle/South Dakota/24-010354-011/2024	100.0	99.3
18	PP753429.1/cattle/Idaho/24-009491-005/2024	100.0	99.3
19	PP755685.1/cattle/Michigan/24-009027-003/2024	100.0	99.3
20	PP755861.1/cat/New Mexico/24-009116-002/2024	100.0	99.4
21	PP753621.1/cattle/Ohio/24-009586-007/2024	100.0	99.4
22	PP756085.1/cattle/North Carolina/24-010327-003/2024	100.0	99.4
23	PP755589.1/cattle/Texas/24-008749-003/2024	100.0	99.4
24	PP692142.1/cat/Texas/24-029329-01/2024	100.0	99.4
25	PQ834101.1/chicken/USA/24-030010-001-original/2024	100.0	99.4
26	PV242322.1/chicken/Germany/2025AI00862/2025	100.0	94.3
27	PV187843.1/chicken/Germany/2024AI05149/2024	100.0	95.3
28	LC718330.1/chicken/Japan/TU20-28,29/2022	100.0	93.9
29	PQ885590.1/human/USA/124/2024	100.0	94.5
30	PV344195.1/human/USA/06-1/2025	100.0	94.5
31	PV289776.1/cat/USA/2/2025	100.0	97.1
32	PV214583.1/cat/USA/25-000089-001-original/2024	100.0	97.1
33	PV396827.1/swine/USA/24-031570-032/2024	100.0	97.2
34	PV520602.1/cattle/USA/WD-210/2025	100.0	97.2
35	PV219153.1/chicken/USA/25-001531-001-original/2025	100.0	97.2
36	PQ885521.1/human/USA/254/2024	100.0	94.7
37	PV494069.1/chicken/USA/25-004736-001-original/2025	100.0	97.4
38	OR388761.1/cat/South Korea/SNU1/2023	100.0	94.1
39	PV388362.1/chicken/Bangladesh/CE-115-09-CA-21-BR-O/2024	100.0	96.8
40	KP638560.1/human/Vietnam/36285/2010	100.0	86.5
41	CY095701.1/chicken/Vietnam/TMU025/2009	100.0	86.3
42	KC784946.1/human/Jiangsu/6/2008	99.9	90.9
43	KC683523.1/swine/Jiangsu/2/2009	99.9	90.5
44	GU182182.1/human/China/6-69/2008	99.9	91

45	FJ492883.1/human/Fujian/1/2007	99.9	91.3
46	HM172096.1/chicken/Fujian/1/2007	100.0	90.8
47	CY098641.1/human/Hubei/1/2006	100.0	90.6
48	HM172069.1/chicken/Sichuan/81/2005	100.0	87.7
49	EU263981.1/human/China/GD02/2006	99.9	91.4
50	DQ371930.1/human/Guangxi/1/2005	99.9	91.4
51	GU052486.1/chicken/Hong Kong/D-06-0947/2006	100.0	91.4
52	EU930996.1/chicken/Vietnam/216/2005	100.0	91.4
53	ON533567.1/equine/China/61/2010	100.0	91.4
54	ON533591.1/equine/China/67/2010	100.0	91.4
55	ON533583.1:/equine/China/68/2010	100.0	91.4
56	ON533575.1/equine/China/25/2010	100.0	91.5
57	KR987709.1/swine/Tabanan/061/2006	100.0	86.9
58	HM440059.1/swine/Banten/UT3062/2005	100.0	87.3
59	HM440051.1/swine/Banten/UT2071/2005	99.9	89.7
60	CY116662.1/cat/Indonesia/5-F2/2005	100.0	86.7
61	EU146681.1/human/Indonesia/283H/2006	100.0	86.5
62	KR732514.1/chicken/West Java/TASIKO	100.0	86.8
63	CY048619.1/chicken/Nigeria/08RS848-94/2007	99.9	89.4
64	EU420046.1/dog/Shandong/sd2/2005	100.0	85.7
65	EU420038.1/dog/Shandong/sd1/2005	100.0	85.7
66	GU052097.1/human/Hong Kong/483/1997	100.0	86.4
67	AY646424.1/swine/Shandong/2/03	100.0	85
68	DQ432037.1/swine/Fujian/2001	99.9	89.5
69	AY747609.1/swine/Fujian/1/2003	100.0	86.4
70	KX364460.1/swine/Shandong/SD1/2014	99.9	89.6
71	EF456799.1/human/Vietnam/JP14/2005	99.9	89.4
72	HM114521.1/human/Vietnam/HN30262IIIM3/2004	99.8	89.4
73	EF541410.1/human/Thailand/SP83/2004	100.0	87.7
74	AY649382.1/chicken/Thailand/CH-2/2004	99.9	89.4
75	MG668907.1/human/Thailand/NBL1/2006	100.0	89.5
76	DQ530173.1/dog/Thailand/KU-08/04	99.8	89.5
77	DQ236077.1/cat/Thailand/KU-02/04	99.8	89.7
78	AB450558.1/chicken/Thailand/NIAH6604/2004	99.8	89.70%

Table S6: NP gene sequences of 62 isolates of H5N1 clade 2.3.4.4b

S.No.	Sequence detail (GenBank ID/Host/Place/Isolate ID/Year)	Coverage (%)	Identity (%)
1.	PV124897.1/cattle/ Hong Kong/RG-DelNS1-p10/2024	100%	100%
2.	PV647684.1/cattle/Colorado/H5N1_CO_Milk/2024	99.20%	83.40%
3.	PP755473.1/goat/Minnesota/24-007234-059/2024	95.70%	82.60%
4.	PQ834190.1/goat/MN/24-007234-044/2024	95.70%	82.60%
5.	OQ683483.1/chicken/Colombia/3499/2022	100.00%	83.10%
6.	PQ295908.1/human/Chile/25945/2023	95.70%	82.30%
7.	PV289777.1/cat/USA/2/2025	95.70%	83.20%
8.	PV344197.1/human/Ohio/06-1/2025	98.40%	83.00%
9.	PV649620.1/chicken/NY/25-010935-007-original/2025	95.70%	83.00%

10.	PV612031.1/human/Canada/PHL-2032-recombinant/2025	95.70%	83.00%
11.	PV602016.1/swine/Kansas/ExpPig61-NS5DPI/2024	97.40%	82.50%
12.	PP756008.1/feline/USA/24-009311-006/2024	95.70%	82.60%
13.	PV601992.1/cattle/Kansas/Inoc-TX-24-029328-01/2024	97.50%	82.60%
14.	PV133378.1/cattle/Texas/A241900097-37/2024	95.70%	82.50%
15.	PQ863964.1/human/California/216/2024	98.40%	82.50%
16.	PV572346.1/feline/WA/25-007097-001-original/2025	95.70%	82.60%
17.	CY029776.1/chicken/Malaysia/5223/2007	95.30%	82.30%
18.	KF735638.1/chicken/Laos/LH2/2010	95.70%	82.80%
19.	KP638532.1/human/Vietnam/UT36282/2010	99.80%	82.70%
20.	KU971359.1/chicken/Ghana/15VIR2588-10/2015	95.70%	83.20%
21.	KY926748.1/chicken/Cameroon/16VIR3791-12/2016	95.70%	83.40%
22.	MF112733.1/chicken/Nigeria/16VIR5840-92/2016	95.70%	83.20%
23.	MF969273.1/swine/Zhejiang/SW57/2015	95.70%	83.40%
24.	KY614952.1/chicken/Indonesia/04160512/2016	100.00%	83.80%
25.	KX247642.1/human/Egypt/MOH-NRC-8434/2014	100.00%	82.00%
26.	CY088770.1/human/Bangladesh/3233/2011	95.70%	82.50%
27.	EU574922.1/chicken/Israel/1055/2008	95.70%	82.50%
28.	EF605600.1/chicken/Russia/2/2007	98.40%	82.70%
29.	CY016790.1/chicken/Afghanistan/1207/2006	97.30%	82.60%
30.	CY037771.1/chicken/Pakistan/NARC3303.4/2006	100.00%	83.10%
31.	CY029997.1/chicken/Kuwait/KISR9/2007	98.00%	82.60%
32.	FM163441.1/chicken/Poland/R3248/2007	100.00%	83.20%
33.	CY020664.1/chicken/Sudan/1784-8/2006	97.30%	82.60%
34.	EU277836.1/chicken/Burkina Faso/1347-16/2006	97.30%	82.90%
35.	CY021520.1/chicken/Ivory Coast/1787-35/2006	97.30%	82.70%
36.	KF001503.1/chicken/Cambodia/X0305302/2013	98.70%	83.00%
37.	KF001373.1/human/Cambodia/X0123311/2013	98.20%	83.30%
38.	KP336346.1/chicken/Nepal/08TI86/2013	100.00%	82.60%
39.	KX215429.1/chicken/Bhutan/1029/2012	100.00%	82.60%
40.	LC208489.1/chicken/Japan/AQ-HE79/2015	99.20%	83.30%
41.	MF598580.1/human/Nanjing/1/2015	95.70%	83.60%
42.	JN808018.1/chicken/Korea/IC546/2011	95.70%	83.40%
43.	CY014254.1/feline/Indonesia/CDC1/2006	95.70%	82.90%
44.	JX235400.1/human/Indonesia/UT3006/2005	95.70%	82.90%
45.	HM440094.1/swine/Indonesia/UT6003/2006	95.70%	83.20%
46.	DQ530174.1/dog/Thailand/KU-08/2004	96.10%	82.40%
47.	DQ236082.1/feline/Thailand/KU-02/2004	95.70%	82.70%
48.	HM590798.1/chicken/Thailand/ICRC-7372/2010	96.20%	82.70%
49.	HM440054.1/swine/Indonesia/UT2071/2005	95.70%	83.20%
50.	ON533578.1/equine/China/25/2010	95.70%	83.20%
51.	ON533586.1/equine/China/68/2010	95.70%	82.90%
52.	CY015084.1/chicken/Scotland/1959	100.00%	84.40%
53.	PQ278126.1/chicken/China/Cangzhou03/2023	98.70%	83.60%
54.	PV388361.1/chicken/Bangladesh/CE-115-09-CA-21-BR-O/2024	95.70%	83.40%

55.	PP732686.1/chicken/Philippines/BA-MHN/2022	98.70%	83.70%
56.	OR680858.1/feline/South Korea/SNU2/2023	97.90%	83.90%
57.	PV410637.1/chicken/ Viet Nam/LBFecal247MC/2023	100.00%	82.50%
58.	PQ109034.1/chicken/India/240008/2024	95.70%	83.20%
59.	PV242323.1/chicken/Germany-NI/2025AI00862/2025	98.00%	83.60%
60.	PV033330.1/human/England/0480160/2025	95.70%	83.20%
61.	OR783370.1/chicken/Egypt/BA20355C/2022	100.00%	83.30%
62.	OQ632899.1/chicken/France/21328/2021	95.70%	83.20%

Table S7: NA gene sequences of 61 isolates of H5N1 clade 2.3.4.4b

S.No.	Sequence detail (GenBank ID/Host/Place/Isolate ID/Year)	Coverage (%)	Identity (%)
1.	PV649629.1/chicken/NY/25-010935-008-original/2025	100.00	100.00
2.	PQ885592.1/human/Iowa/124/2024	100.00	97.60
3.	PV396829.1/pig/OR/24-031570-032/2024	100.00	99.30
4.	PQ809557.1/human/Louisiana/12/2024	100.00	97.80
5.	PV410638.1/chicken/Viet Nam/LBFecal247MC/2023	100.00	82.10
6.	OQ547321.1/chicken/Peru/AIS0547/2022	100.00	84.10
7.	OR910233.1/chicken/Atacama/235254-6/2023	100.00	82.80
8.	OR381610.1/chicken/Uruguay/047-M1/2023	100.00	85.40
9.	PP752489.1/chicken/Minnesota/24-005907-001/2024	100.00	85.00
10.	PP755360.1/goat/Minnesota/24-007234-003/2024	100.00	85.00
11.	PQ834215.1/goat/MN/24-007234-050/2024	100.00	85.00
12.	PP752561.1/goat/Minnesota/24-007234-037/2024	100.00	84.90
13.	PV649541.1/cattle/ID/25-010523-001-original/2025	100.00	85.00
14.	PP752695.1/cat/Montana/24-009038-001/2024	100.00	85.20
15.	PV649093.1/cat/CA/25-011002-002-original/2025	100.00	84.90
16.	PP753383.1/cattle/Texas/24-009367-001/2024	100.00	85.10
17.	PP756063.1/cattle/North Carolina/24-010327-002/2024	100.00	85.20
18.	PP755935.1/cattle/New Mexico/24-009306-004/2024	100.00	85.30
19.	PP737541.1/cattle/Ohio/B24OSU-UW11-863/2024	100.00	85.20
20.	PQ051492.1/cattle/IA/24-016252-001-original/2024	100.00	85.20
21.	PV124885.1/cattle/Hong Kong/RG-DelNS1-p1/2024	100.00	82.40
22.	PP755687.1/cattle/Michigan/24-009027-003/2024	100.00	85.20
23.	PV602017.1/swine/Kansas/ExpPig61-NS5DPI/2024	100.00	84.30
24.	PV242364.1/chicken/Germany/2025AI01260/2025	94.80	85.20
25.	OR388765.2/cat/South Korea/SNU1/2023	100.00	82.90
26.	PP732685.1/chicken/Philippines/BA-MHN/2022	100.00	83.70
27.	PV344196.1/human/Ohio/06-1/2025	100.00	83.40
28.	PQ685078.1/chicken/Egypt/BA20355C/2022	100.00	82.80
29.	PQ109035.1/chicken/India/240008/2024	100.00	85.00
30.	PV335482.1/chicken/Bangladesh/63995/2024	95.70	77.50
31.	DQ997333.1/chicken/Jilin/hl/2004	100.00	82.30
32.	AY747610.2/swine/Fujian/1/2003	100.00	82.90
33.	HM440053.1/swine/Banten/UT2071/2005	95.70	80.40
34.	HM440093.1/swine/East Java/UT6003/2006	95.70	80.20
35.	HM440141.1/swine/South Kalimantan/UT6015/2006	95.70	80.30

36.	CY116648.1/human/Indonesia/5/2005	95.70	77.40
37.	KC831475.1/dog/Wonogiri/BBVW-275/2007	95.50	79.20
38.	HM172165.1/human/Anhui/2/2005	95.70	77.90
39.	ON533568.1/equine/China/61/2010	95.70	80.80
40.	ON533576.1/equine/China/25/2010	95.70	80.70
41.	GU371912.1/equine/Egypt/av1/2009	89.90	78.20
42.	DQ236078.1/cat/Thailand/KU-02/04	95.70	79.50
43.	AY577315.3/human/Thailand/3(SP-83)/2004	95.70	77.70
44.	EF456801.1/human/Vietnam/JP14/2005	95.70	80.60
45.	FJ225473.1/human/Cambodia/R0405050/2007	95.70	78.00
46.	KC683526.1/swine/Jiangsu/1/2008	95.70	80.10
47.	AB462297.1/human/Shanghai/1/2006	95.70	77.80
48.	DQ835315.1/human/China/GD01/2006	95.70	77.80
49.	DQ997077.1/swine/Anhui/cb/2004	95.70	77.70
50.	KJ816935.1/swine/Shandong/SD1/2013	95.70	78.00
51.	KX364467.1/swine/Shandong/SD2/2014	95.70	80.50
52.	EF587279.1/human/Beijing/01/2003	95.70	78.20
53.	DQ997263.1/swine/Guangxi/wz/2004	95.70	78.10
54.	EU420040.1/dog/Shandong/sd1/2005	95.70	78.00
55.	CY062438.1/human/Egypt/5614-NAMRU3/2006	95.10	80.60
56.	DQ835390.1/cat/Iraq/820NAMRU3/2006	95.70	80.40
57.	EU146885.1/human/Iraq/1/2006	95.70	80.60
58.	DQ643984.1/cat/Germany/606/2006	95.70	77.90
59.	EF205168.1/chicken/Suzdalka/06/05	95.70	78.00
60.	CY098663.1/human/Xinjiang/1/2006	95.70	80.40
